# Disruption of gray matter morphological networks in patients with paroxysmal kinesigenic dyskinesia

**DOI:** 10.1002/hbm.25230

**Published:** 2020-10-15

**Authors:** Xiuli Li, Du Lei, Running Niu, Lei Li, Xueling Suo, Wenbin Li, Chen Yang, Tianhua Yang, Jiechuan Ren, Walter H. L. Pinaya, Dong Zhou, Graham J. Kemp, Qiyong Gong

**Affiliations:** ^1^ Huaxi MR Research Center (HMRRC), Department of Radiology West China Hospital of Sichuan University Chengdu Sichuan Province China; ^2^ Department of Radiology Sichuan Cancer Hospital & Institute, Sichuan Cancer Center, School of Medicine, University of Electronic Science and Technology of China Chengdu China; ^3^ Department of Psychiatry and Behavioral Neuroscience University of Cincinnati Cincinnati Ohio USA; ^4^ Department of Neurology West China Hospital of Sichuan University Chengdu Sichuan Province China; ^5^ Department of Psychosis Studies Institute of Psychiatry, Psychology & Neuroscience, King's College London London UK; ^6^ Center of Mathematics, Computing, and Cognition Universidade Federal do ABC Santo André Brazil; ^7^ Liverpool Magnetic Resonance Imaging Centre (LiMRIC) and Institute of Life Course and Medical Sciences, University of Liverpool Liverpool UK; ^8^ Research Unit of Psychoradiology Chinese Academy of Medical Sciences Chengdu China; ^9^ Functional and Molecular Imaging Key Laboratory of Sichuan University Chengdu China

**Keywords:** gray matter networks, machine learning, paroxysmal kinesigenic dyskinesia, structural MRI, topological organization

## Abstract

This study explores the topological properties of brain gray matter (GM) networks in patients with paroxysmal kinesigenic dyskinesia (PKD) and asks whether GM network features have potential diagnostic value. We used 3D T1‐weighted magnetic resonance imaging and graph theoretical approaches to investigate the topological organization of GM morphological networks in 87 PKD patients and 115 age‐ and sex‐matched healthy controls. We applied a support vector machine to GM morphological network matrices to classify PKD patients versus healthy controls. Compared with the HC group, the GM morphological networks of PKD patients showed significant abnormalities at the global level, including an increase in characteristic path length (*Lp*) and decreases in local efficiency (*E*_loc_), clustering coefficient (*Cp*), normalized clustering coefficient (*γ*), and small‐worldness (*σ*). The decrease in *Cp* was significantly correlated with disease duration and age of onset. The GM morphological networks of PKD patients also showed significant changes in nodal topological characteristics, mainly in the basal ganglia‐thalamus circuitry, default‐mode network and central executive network. Finally, we used the GM morphological network matrices to classify individuals as PKD patients versus healthy controls, achieving 87.8% accuracy. Overall, this study demonstrated disruption of GM morphological networks in PKD, which might extend our understanding of the pathophysiology of PKD; further, GM morphological network matrices might have the potential to serve as network neuroimaging biomarkers for the diagnosis of PKD.

## Graphical abstract

This study explores the topological properties of brain gray matter (GM) networks in patients with paroxysmal kinesigenic dyskinesia (PKD), providing the first demonstration that PKD features disrupted topological organization in GM morphological networks and the GM morphological network matrices might have potential as neuroimaging biomarkers to assist in clinical PKD diagnosis.

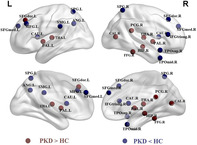

## 1 INTRODUCTION

Paroxysmal kinesigenic dyskinesia (PKD) is a rare movement disorder characterized by transient and recurrent dystonic or choreoathetoid attacks, mainly triggered by sudden voluntary movement (Bruno et al., 2004). According to etiology, PKD can be divided into idiopathic (also called primary) PKD and secondary disease (Waln & Jankovic, 2015). Idiopathic PKD does not show any abnormalities on conventional neuroimaging or electroencephalography (EEG) between attacks. Secondary PKD is caused by various neurological and medical diseases such as multiple sclerosis and stroke affecting the basal ganglia.

Previous studies have revealed a complex pattern of abnormalities affecting diverse subcortical regions including the basal ganglia and thalamus (Joo et al., 2005; Kim, Kim, Kim, Suh, & Koh, 2015; Shirane, Sasaki, Kogure, Matsuda, & Hashimoto, 2001; Zhou, Chen, Gong et al., 2010; Zhou, Chen, Zhang, et al., 2010), as well as cortical regions including the motor cortex, somatosensory cortex, presupplementary motor area and right inferior frontal gyrus (Hsu, Kwan, et al., 2013; Hsu, Liao, et al., 2013; H. F. Li et al., 2019; Liu et al., 2018) in PKD. However, the pathophysiological mechanisms in PKD are not fully understood.

The rapid development of psychoradiology has advanced our understanding of the complex brain alterations in patients with neuropsychiatric disorders (Gong, 2020). Analyzing the structural networks which form the anatomical and physiological substrate that shapes brain function (Qi, Meesters, Nicolay, Ter Haar Romeny, & Ossenblok, 2016; Sporns, Tononi, & Kotter, 2005) can assist our better understanding of brain abnormalities. Currently, there are two modalities available for imaging structural connectivity in humans: tractography using diffusion tensor imaging (DTI) and structural covariance network (SCN) analysis based on structural T1‐weighted MRI sequences (Seidlitz et al., 2018; Sun et al., 2018). These two approaches investigate different mechanisms of human network organization. The former seeks to reconstruct anatomical connectivity from bundles of nerve fibers in white matter. The latter aims to construct structural networks by calculating interregional morphological associations based on the structural covariance of gray matter volume and cortical thickness (Alexander‐Bloch, Giedd, & Bullmore, 2013; He, Chen, & Evans, 2007). Analyses of structural covariance focus on the covariation (i.e., the correlation) in structural markers between different brain regions, and can offer more insights into their topographical organization. It is increasingly apparent that the study of SCNs is a robust and valuable tool for investigating topological organization in brain disorders (T. Chen et al., 2017; Niu et al., 2018; Tijms, Series, Willshaw, & Lawrie, 2012; Tijms et al., 2014) and can provide information complementary to other connectivity approaches, such as resting‐state fMRI and diffusion imaging. In the present study, we focused on gray matter (GM) SCNs (i.e., morphological networks).

We used a new method proposed by Kong and colleagues (Kong et al., 2015; Kong et al., 2014; H. Wang, Jin, Zhang, & Wang, 2016) to construct individual morphological networks. In this method, networks are constructed with nodes representing small brain regions whose connections are computed by evaluating intracortical similarities in GM morphological distributions, which not only quantify the interregional relations within each participant's brain but also take into account the structural complexity of the cerebral cortex. We used this method to investigate the topological organization of brain GM networks in PKD, in order to throw light on the pathophysiology of PKD from a GM network perspective. Moreover, to explore their potential as an aid to clinical diagnosis, we used the extracted GM morphological network matrices of GM networks as features to classify individual patients with PKD versus healthy controls.

Based on the finding of altered topological organization of functional brain networks in drug‐naïve patients with PKD (Y. Zhang et al., 2020), we hypothesized (i) that PKD patients would show disrupted topological organization in their GM morphological networks, and that these disruptions would be associated with the clinical characteristics. In addition, as previous studies have revealed focal abnormalities in the basal ganglia and thalamus in PKD (Joo et al., 2005; Kim et al., 2015; Zhou, Chen, Gong et al., 2010), we also hypothesized (ii) that significantly altered nodal topological properties would be observed in these regions. Moreover, many studies have shown that network‐based biomarkers, which can capture the brain network structure in a phenotype and help to elucidate the role of known subsystems, have diagnostic potential in brain disorders (Sacchet, Prasad, Foland‐Ross, Thompson, & Gotlib, 2015; Wen et al., 2017). We therefore hypothesized (iii) that GM morphological network matrices could serve as neuroimaging biomarkers to detect PKD with significant accuracy.

## 2 MATERIALS AND METHODS

### 2.1 Participants

Ninety patients with idiopathic PKD were recruited from the Movement Disorders Outpatient Clinic of West China Hospital, Sichuan University. The diagnosis of idiopathic PKD was made according to Bruno's diagnostic criteria (Bruno et al., 2004), as follows: (i) identified kinesigenic trigger for the attacks; (ii) short duration of attacks (1 min); (iii) no pain or loss of consciousness during attacks; (iv) normal neurologic examination and exclusion of other organic diseases; (v) control of attacks with phenytoin or carbamazepine, if tried; and (vi) age at onset between 1 and 20 years if no family history of PKD.

A total of 120 age‐ and sex‐matched HCs were recruited from the local area through poster advertisements. The exclusion criteria for all participants were as follows: (i) presence of focal brain lesions on routine MRI; (ii) claustrophobia or MRI incompatibility; (iii) history of alcohol/substance abuse; (iv) comorbidity with neurological or psychiatric disorders or serious physical disease (including traumatic brain injury, cerebrovascular disease, hypertension, diabetes mellitus, ischemic heart disease, chronic liver disease, or other chronic systemic disorders); and (v) presence of head movement artifacts on scanning. Eighty‐seven patients with PKD and 115 HCs were finally selected for the study. All participants' clinical information was obtained according to a standard protocol. To control for possible confounding effects of antiepileptic drugs (AEDs), we recruited only patients who had been on medication for no more than 3 months, and patients who had previously taken medication intermittently but not within 6 months before the study. Additionally, AED‐treated patients were scanned only after medication had been withdrawn for at least 12 hours. The patients and controls were recruited from July 2013 to June 2018. Written informed consent was obtained from all participants or their legal guardians. The study was approved by the local human research ethics committee.

### 2.2 Data acquisition

All patients and controls were scanned on the same instrument (3.0 T Siemens Trio, Erlangen, Germany) with the same sequence and a 12‐channel head coil. Foam padding was used to minimize head motion. High‐resolution 3D T1‐weighted images were acquired using a magnetization‐prepared rapid gradient‐echo sequence with the following parameters: repetition time, 1,900 ms; echo time, 2.26 ms; inversion time, 900 ms; flip angle, 9°; field of view, 256 × 256 mm^2^; matrix size, 256 × 256; slice thickness, 1 mm; no interslice gap; voxel size, 1 × 1 × 1 mm^3^; number of slices, 176. The total acquisition time was 420 s.

### 2.3 Data preprocessing

Structural images were preprocessed using voxel‐based morphometry (VBM) implemented in Statistical Parametric Mapping (SPM) version 12 (http://www.fil.ion.ucl.ac.uk/spm/software/spm12/). VBM is an automatic whole‐brain neuroimaging analysis technique that allows the quantification of local morphological features from individual MRI data (Ashburner & Friston, 2000). First, the MRI data for each participant were manually assessed by two experienced radiologists/investigators to exclude scanning artifact. Second, the individual structural data were segmented to obtain the GM images using the unified segmentation tool (Ashburner & Friston, 2005) in SPM 12. Next, the GM images were nonlinearly coregistered using Diffeomorphic Anatomical Registration Through Exponentiated Lie Algebra (DARTEL) (Ashburner, 2007), which involves the iterative calculation of a study‐specific template based on the GM images from all participants, then warping participants' GM images into the generated template. Then the GM images were normalized to the standard Montreal Neurological Institute (MNI) space to yield GM images in the same space as the brain parcellation. Thereafter, in order to preserve tissue volume following warping, voxel values in individual GM images were modulated by multiplying the Jacobian determinants derived from the normalization. Then all modulated GM images were resampled to 2 mm^3^ voxels and individually smoothed with a 6 mm full‐width at half‐maximum (FWHM) Gaussian kernel. Finally, the smoothed and modulated GM images, comprising morphological volume information for each voxel, which was comparable across participant, were obtained for further analyses.

### 2.4 Construction of individual GM morphological networks

**FIGURE 1:**
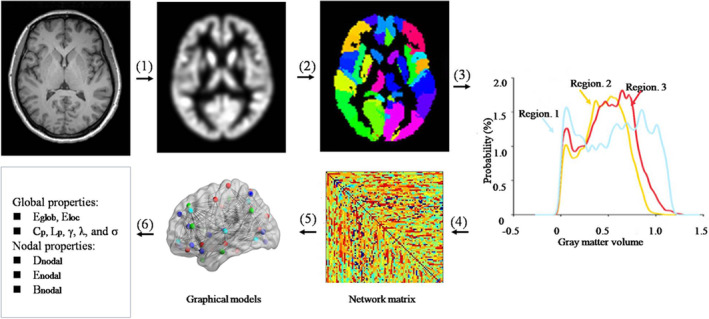
A flowchart for the construction of gray matter (GM) morphological networks using T1‐weighted MRI. (1) Individual structural images were segmented, normalized, modulated, and smoothed using a routine VBM procedure to obtain the GM maps. (2) The GM map was divided into 90 regions according to the AAL atlases. (3) For each region, its GM volume was extracted and used to estimate the PDF (4) The KLS between the PDFs of each pair of regions was calculated, resulting in a similarity matrix. (5) Individual brain networks were represented as graphs. (6) Finally, the network properties were calculated at both the global and nodal levels

The pivotal task in human brain network construction is to define the nodes and edges. In the present GM structural network, nodes were defined as brain regions using the automated anatomical labeling (AAL) algorithm, which parcellates the whole GM into 90 anatomical regions of interest (ROIs) (Tzourio‐Mazoyer et al., 2002). Edges were defined as the statistical similarity of morphological distributions between different brain regions and were quantified by a Kullback–Leibler divergence‐based similarity (KLS) measure (Kong et al., 2014). The connection matrices for each subject's network were quantified as follows. First, GM volume values were extracted of all the voxels within each ROI based on the AAL. Then the probability density functions (PDFs) of these values were estimated using kernel density estimation (KDE) (H. Wang et al., 2016). The KDE bandwidths were not set manually but were adaptively estimated from the data using Scott's rule (Scott, 2015). This analysis was performed using public Matlab code provided by Botev (function:kde;http://www.mathworks.com/matlabcentral/fileexchange/14034-kernel-density‐estimator). Next, the Kullback–Leibler (KL) divergence was used to quantify the similarity of PDFs of different brain regions and convert it to a similarity measure. KL divergence is an index from probability theory to measure the difference between two probability distributions; equivalently, from the perspective of information theory it is the information lost when one probability distribution is used to approximate another (Burnhan & Anderson, 2002). The details of the analyses are described elsewhere (Kong et al., 2015; Kong et al., 2014; H. Wang et al., 2016). Finally, we calculated the KL‐based similarity values between all possible pairs of 90 brain regions to generate a 90 × 90 similarity matrix for each subject. In this 90 × 90 network matrix, each row and column represent a brain region, and each element represents the similarity of morphological distributions between brain regions. The range of possible KLS values is 0 to 1, where 1 represents an identical distribution for the two regions. The main analytical steps are shown in Figure 1.

### 2.5 Network properties

The GRETNA toolbox (http://www.nitrc.org/projects/gretna/) in MATLAB was used to calculate the network properties of the brain networks (J. Wang et al., 2015) and individual GM morphological networks were thresholded and weighted. A wide range of sparsity (S) thresholds was applied to all the correlation matrices. The minimum and maximum values of S were determined to ensure that the thresholded networks were estimable for the small‐worldness with sparse properties, and had the minimum number of spurious edges (Watts & Strogatz, 1998). With these limits, the range of our threshold was 0.10 < S < 0.34 with an interval of 0.01. For the brain networks at each sparsity level, we calculated both global and nodal network metrics. Direct comparisons across different levels of sparsity could cause the same network metric to take on different values, which would make the results unstable and inconsistent. For each network metric we therefore calculated the area under the curve (AUC) reflecting measures across the sparsity parameter S, providing a summarized scalar for the topological characterization of brain networks without using an arbitrary single threshold selection. This measure has proven sensitive in detecting topological alterations of brain networks (He et al., 2009; J. Zhang et al., 2011).

Both global and nodal network properties were calculated for the brain networks at each sparsity threshold. At the global level, we focused on two key organizational aspects of the human brain: small‐worldness (Watts & Strogatz, 1998) and network efficiency (Achard & Bullmore, 2007). We examined the following global metrics: the clustering coefficient (*Cp*), characteristic path length (*Lp*), normalized clustering coefficient (*γ*), normalized characteristic path length (*λ*), small‐worldness (*σ*), local efficiency (*E*_loc_), and global efficiency (*E*_glob_). Briefly, *Cp* is defined as the average of the clustering coefficients over all nodes and quantifies the “cliquishness” and reflects the extent of local interconnectivity; *Lp* measures the mean distance or routing efficiency between any pair of nodes in a network, and lower values indicate higher routing efficiency; small‐world attributes (*γ*, *λ*, and *σ*) indicate the degree of small‐world organization, which reflects an optimal balance of integration and segregation for a network; *E*_glob_ measures the global efficiency of parallel information transfer in the network; and *E*_loc_ reflects the network fault tolerance level, the communication efficiency among the first neighbors of a node when it is removed. The nodal topological characteristics, including nodal degree, nodal efficiency, and nodal betweenness centrality, were chosen because these measures are interrelated, each providing a different viewpoint on the major features of the large‐scale network. Nodal degree reflects the capacity to communicate information, nodal efficiency characterizes the efficiency of parallel information transfer, and nodal betweenness centrality captures the influence of a node over information flow between other nodes in the network. Detailed formulae, usages and explanations of these metrics can be found in an excellent methodological review (Rubinov & Sporns, 2010).

### 2.6 Statistical analysis

#### 2.6.1 Comparison of demographic and clinical variables

The analyses of demographic and clinical data were performed with IBM SPSS Statistics V21.0. Two‐tailed independent‐sample *t*‐test and chi‐squared test were used to compare quantitative and qualitative variables.

#### 2.6.2 Comparison of network metrics

We performed nonparametric permutation tests on the AUC of each network metric to assess between‐group differences (Lei et al., 2015; J. Zhang et al., 2011). Briefly, we first computed the between‐group differences using the AUC value of each network metric across *S* values. To correct for multiple comparisons, we randomly reallocated all the values into two groups and recalculated the mean differences between the two randomized groups for each network metric. This randomization procedure was repeated 10,000 times, and the 95th percentiles of each distribution were used as the critical value for significance testing. Furthermore, when node centrality was tested, the Benjamini‐Hochberg procedure was used to correct for multiple comparisons by controlling the false discovery rate (Benjamini, Drai, Elmer, Kafkafi, & Golani, 2001).

#### 2.6.3 Network‐based statistical analysis

Region pairs with between‐group differences of nodal characteristics in patients with PKD were assessed using the network‐based statistics (NBS) (http://www.nitrc.org/projects/nbs/) approach. First, we chose the nodes that exhibited between‐group differences in at least one of the three node centrality measures (nodal degree, efficiency, and betweenness), and then created a connection matrix among those nodes for each participant. Second, the NBS approach was applied to define a set of suprathreshold links that included any connected components (threshold = 2.9, *p* < .05). To estimate the significance for each component, the nonparametric permutation approach (10,000 permutations) was also used. A detailed description of this approach has been previously published (Zalesky, Fornito, & Bullmore, 2010).

#### 2.6.4 Correlation analysis

For all significantly altered global and nodal network properties in the topological analysis, we examined the correlations with clinical variables by partial correlation analysis, controlling for age, sex, and years of education as confounding variables (*p* < .05). Outlier detection was performed to ensure that no outliers were included in the analysis. All statistical analyses were performed using the SPSS software (IBM SPSS Statistics V21.0).

#### 2.6.5 Classification analysis

We determined the efficacy of detecting PKD at the individual level using morphological network matrices. To do this we measured the performance of a support vector machine (SVM) (Cortes & Vapnik, 1995) trained to classify PKD patients versus healthy controls using the GM morphological network matrices (90 × 90 network matrix).

The SVM is a widely used machine learning model, and we used the implementation from the Scikit‐Learn library (Pedregosa et al., 2012) that is based on LIBSVM (Chang & Lin, 2011). It works as follows: first, the model maps the input data from the training set to the feature space using a set of mathematical functions known as kernels. Here, a linear kernel was preferred to a nonlinear kernel to minimize the risk of overfitting. In this feature space, the model learns the optimum separation surface that maximizes the margin between different classes. In our case the linear SVM has one hyperparameter (the soft margin parameter C), which affects the model's training by controlling the trade‐off between reducing training errors and increasing the separation margin. Once the separation surface is determined, it can be used to predict the class of new unseen observations.

To obtain a reliable estimate of the performance of the models, we used a 10‐fold stratified cross‐validation scheme. In this scheme the 202 participants were divided into 10 nonoverlapping partitions, each with the same proportion of patients and healthy controls. In each one of the 10 iterations of the cross‐validation, nine partitions (composed of 181 subjects) were used as the training set to train the SVM model, and then the trained model was used to obtain the predictions in the remaining partition (composed of 21 subjects and called the “test set”). These predictions were used to calculate the performance metrics (balanced accuracy, specificity, and sensitivity), and since the test set was not part of the training process, the resulting values were unbiased. The reported performance in each case is the mean value across the cross‐validation iterations. Finally, the statistical significance was estimated using the permutation method (1,000 permutations).

In each iteration of the cross‐validation, we also performed a nested cross‐validation inside the training set (i.e., 10‐fold stratified nested cross‐validation) to select the optimum C value for the SVM. This parameter was optimized by performing a grid search in the following range of values: *C* = 10^−3^, 10^−2^, 10^−1^, 10^0^, 10^1^, 10^2^, 10^3^, 10^4^. After selecting the best C value based on the balanced accuracy, an SVM was trained using the whole training set and used to assess performance on the test set. Note that the test set was not used during this hyperparameter search, to avoid biased results. The code used is available at http://github.com/Warvito/integrating-multi-modal-neuroimaging.

## 3 RESULTS

### 3.1 Demographic and clinical characteristics

The demographic and clinical data are summarized in Table 1. There were no significant differences in age or sex between patients with PKD and HCs (*p* > .05). Although the mean duration of education was greater in the HC group than in PKD (*p* < .05), we included years of education as a covariate in the correlation analysis between network properties and clinical variables to exclude its possible impact. We also performed Pearson correlation analysis to investigate the relationship between the topological metrics (AUC) and the years of education in both groups, and there were no significant correlations (*p* > .05).

**TABLE 1:** Demographic and clinical characteristics of PKD patients and healthy controls

Characteristics	HC (*n* = 115)	PKD (*n* = 87)	*p* value
Age (y)^a^	22.7 ± 6.2 (10–58)	23.0 ± 9.5 (11–63)	.781^b^
Education (y)^a^	12.9 ± 2.7 (3–19)	11.2 ± 3.2 (4–19)	<.001^b^
Gender (M/F)	95/20	71/16	.854^c^
Age at onset (y)	NA	12.7 ± 5.5 (2–36)	NA
Disease duration (y)^a^	NA	10.0 ± 9.5 (0.2–42)	NA
Family history (+/−)	NA	19/68	NA
Attack frequency (<10/d:>10/d)	NA	61/26	NA
Affected side (L/R/Bil/Alt)	NA	8/11/51/17	NA
Previously treated/drug naïve	NA	81/6	NA
Treatments (OXC/CBZ/others)	NA	24/48/9	NA

*Note:* Data are presented as the means ± standard deviations unless otherwise stated. Parentheses indicate range.

Abbreviations: Alt, alternate; Bil, bilateral; CBZ, carbamazepine; d, day; HC, healthy controls; L, left; NA, not applicable; OXC, oxcarbazepine; PKD, paroxysmal kinesigenic dyskinesia; R, right; y, year.

^a^ Age, disease duration and years of education were defined at the time of MRI scanning.

^b^ Calculated by independent‐sample *t*‐test.

^c^ Calculated by chi‐squared test.

### 3.2 Alterations in global brain network properties

**FIGURE 2:**
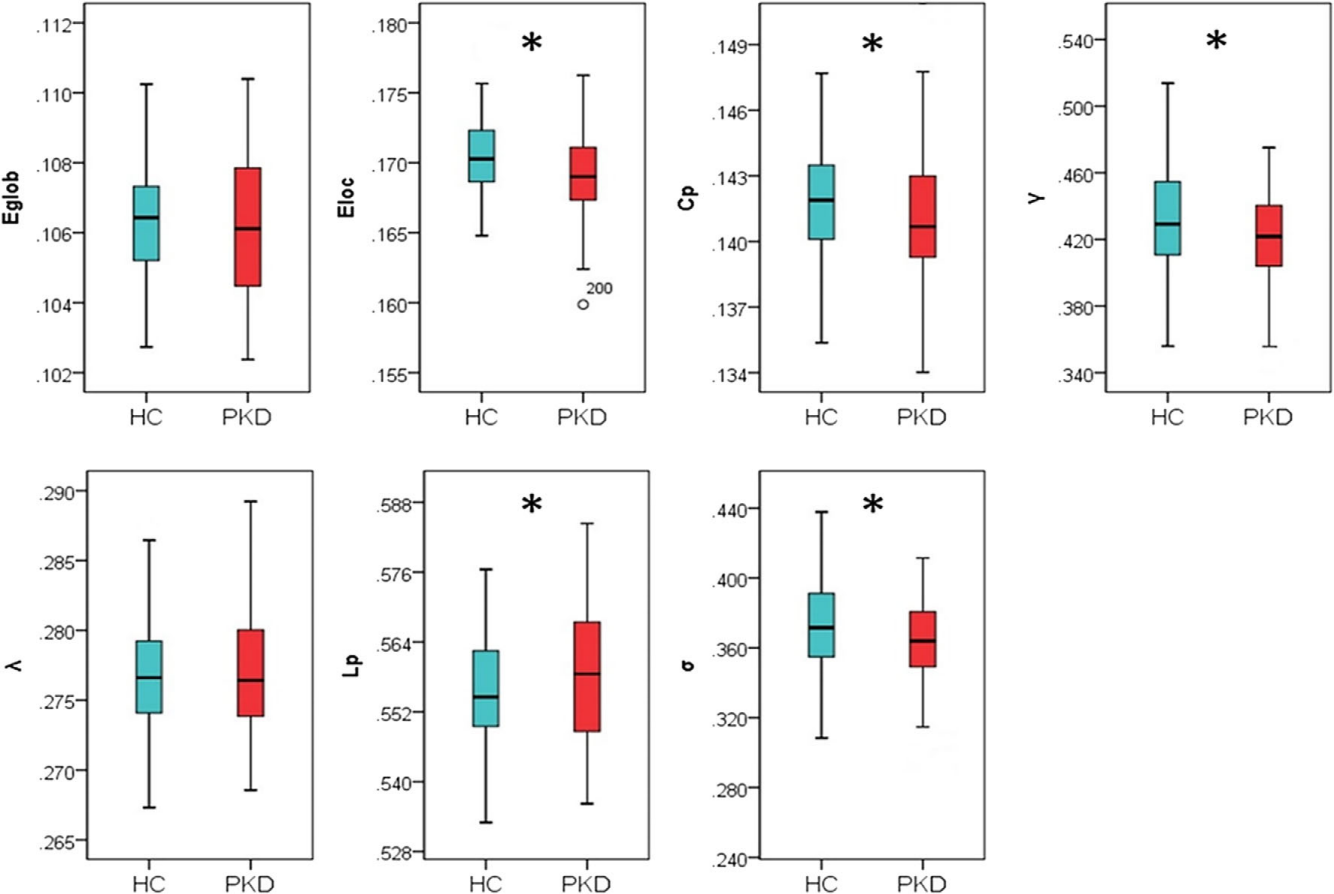
Group differences in global network properties of brain gray matter morphological networks. Relative to healthy controls (HC), patients with paroxysmal kinesigenic dyskinesia (PKD) showed a significant increase in characteristic path length (*Lp*; *p* = .016) and decreases in local efficiency (*E*_loc_; *p* = .001), clustering coefficient (*Cp*; *p* = .030), normalized clustering coefficient (*γ*; *p* = .005), and small‐worldness (*σ*; *p* = .005). There was no significant difference in global efficiency (*E*_glob_; *p* = .238) or normalized characteristic path length (*λ*; *p* = .440). Asterisks designate network metrics showing a significant difference (*p* < .05)

In the defined threshold range, both the PKD and control groups exhibited normalized *Cp* values substantially greater than 1, and normalized *Lp* values approximately equal to 1 (Figure S1), indicating that both groups exhibited the typical features of small‐world topology in the GM morphological network. However, this topology was altered in the PKD patients. Compared with HCs, the PKD patients showed a significant increase in *Lp* (*p* = .016) and decreases in *E*_loc_ (*p* = .001), *Cp*(*p* = .030), *γ* (*p* = .005) and *σ* (*p* = .005). No significant difference was identified in *E*_glob_ (*p* = .238) or *λ* (*p* = .440; Figure 2).

### 3.3 Alterations in nodal brain network properties

**FIGURE 3:**
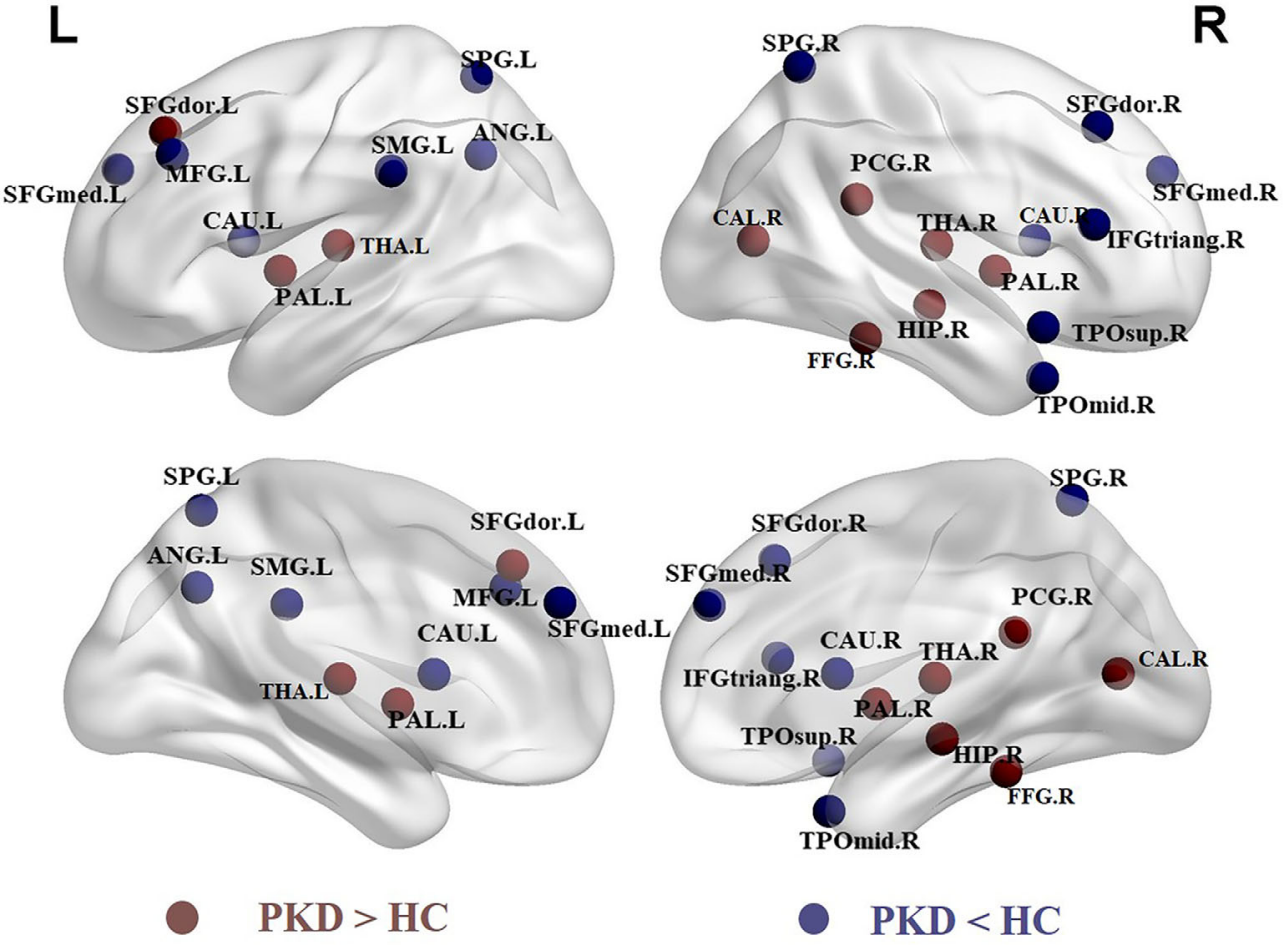
Regions of significant differences in nodal centralities of brain gray matter morphological networks between PKD patients and healthy controls, showing both increases (red) and decreases (blue) in PKD compared to HC. ANG, angular gyrus; CAL, calcarine fissure and surrounding cortex; CAU, caudate; FFG, fusiform gyrus; HC, healthy controls; HIP, hippocampus; IFGtriang, inferior frontal gyrus, triangular part; L, left; MFG, middle frontal gyrus; PAL, pallidum; PCG, posterior cingulate gyrus; PKD, paroxysmal kinesigenic dyskinesia; R, right; SFGdor, superior frontal gyrus, dorsolateral; SFGmed, superior frontal gyrus, medial; SMG, supramarginal gyrus; SPG, superior parietal gyrus; THA, thalamus; TPOmid, temporal pole, middle temporal gyrus; TPOsup, temporal pole, superior temporal gyrus; The results were visualized using the BrainNet viewer package (http://www.nitrc.org/projects/bnv)

Table 2 lists brain regions exhibiting significant between‐group differences in at least one nodal metric in the PKD patients compared to the HCs (false discovery rate corrected, *p* < .05). Among these regions, 6 were located in the subcortical cortices, 6 were in the frontal cortex, 4 were in the parietal cortex, and the others were in the limbic, temporal, and occipital cortices (Figure 3).

**TABLE 2:** Regions showing altered node centrality in PKD patients and healthy controls

Brain regions	Category	*p values*		
		Nodal betweenness	Nodal degree	Nodal efficiency
*PKD > HC*				
SFGdor.L	CEN	.0001*	.3996	.0586
PCG.R	DMN	.0775	.0737	.0008*
HIP.R	DMN	<.0001*	.0377	.4324
CAL.R	Others	.0089	.0034*	.0056*
FFG.R	Others	.0427	.0007*	.0004*
PAL.L	Basal ganglia	.0031*	<.0001*	<.0001*
PAL.R	Basal ganglia	.1807	.0604	<.0001*
THA.L	Thalamus	<.0001*	.0301	.0274
THA.R	Thalamus	.0587	.0092*	.0182
*PKD < HC*				
SFGdor.R	CEN	.1270	.0206	.0026*
MFG.L	CEN	.4130	.0042*	.0128
IFGtriang.R	CEN	.4485	<.0001*	<.0001*
SFGmed.L	DMN	.1738	.0003*	.0002*
SFGmed.R	DMN	.0681	.0020*	.0067*
SPG.L	CEN	.2114	.0001*	.0002*
SPG.R	CEN	.2263	.0006*	<.0001*
SMG.L	CEN	.0005*	.0050*	.0053*
ANG.L	DMN	.1828	.0072*	.0069*
CAU.L	Basal ganglia	.0111	<.0001*	<.0001*
CAU.R	Basal ganglia	.4246	<.0001*	<.0001*
TPOsup.R	DMN	.0048	<.0001*	<.0001*
TPOmid.R	Others	.2262	<.0001*	<.0001*

*Note:* These regions exhibited significant between‐group differences in at least one node centrality parameter (marked by asterisk). Benjamini‐Hochberg false discovery rate correction was applied to each nodal measure. All *p* values were obtained using a permutation test. All the brain regions were defined by AAL (automated anatomical labeling).

Abbreviations: ANG, angular gyrus; CAL, calcarine fissure and surrounding cortex; CAU, caudate; CEN, central executive network; DMN, default‐mode network; FFG, fusiform gyrus; HIP, hippocampus; HC, healthy controls; IFGtriang, inferior frontal gyrus, triangular part; L, left; MFG, middle frontal gyrus; PAL, pallidum; PCG, posterior cingulate gyrus; R, right; SFGdor, superior frontal gyrus, dorsolateral; SFGmed, superior frontal gyrus, medial; SMG, supramarginal gyrus; SPG, superior parietal gyrus; THA, thalamus; TPOmid, temporal pole, middle temporal gyrus; TPOsup, temporal pole, superior temporal gyrus.

### 3.4 NBS results

**FIGURE 4:**
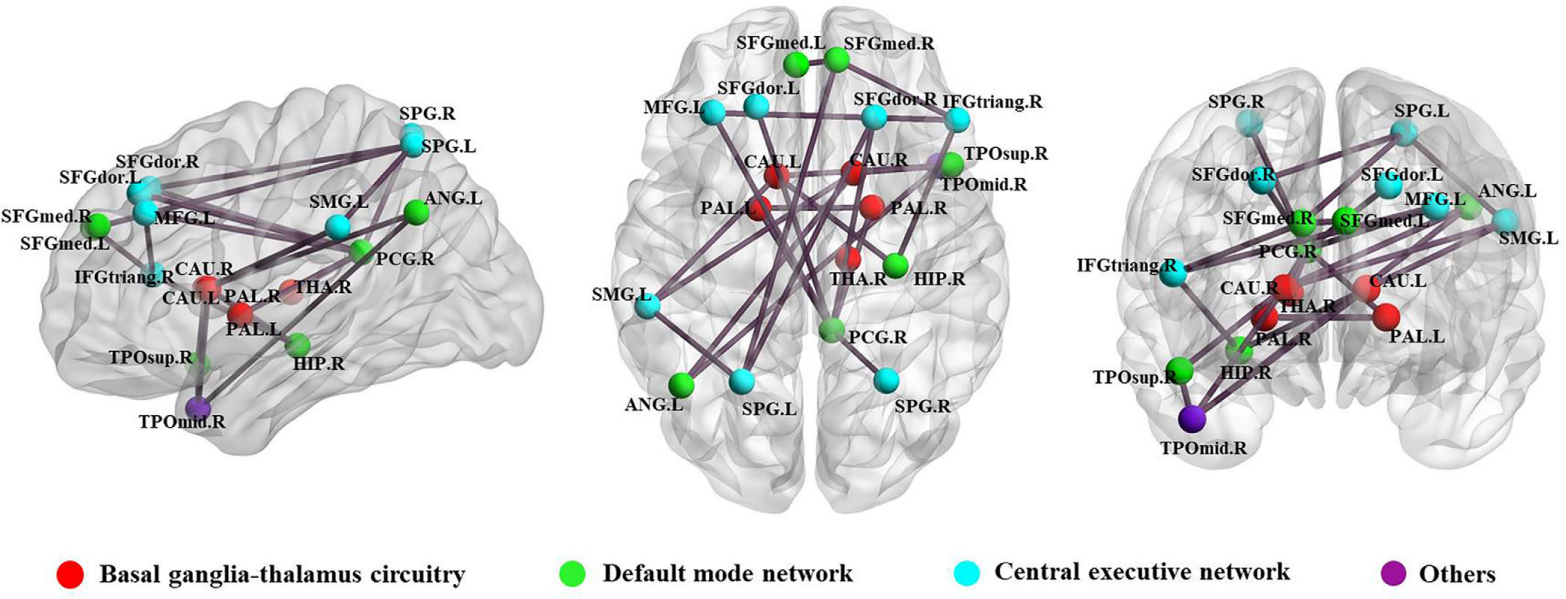
Regions exhibiting altered node centrality and decreased structural connections in PKD patients compared to healthy controls. These connections formed a single network with 19 nodes and 24 edges (*p* < .05, corrected). ANG, angular gyrus; CAU, caudate; HIP, hippocampus; IFGtriang, inferior frontal gyrus, triangular part; L, left; PAL, pallidum; PCG, posterior cingulate gyrus; R, right; SFGdor, superior frontal gyrus, dorsolateral; SFGmed, superior frontal gyrus, medial; SMG, supramarginal gyrus; SPG, superior parietal gyrus; THA, thalamus; TPOmid, temporal pole, middle temporal gyrus; TPOsup, temporal pole, superior temporal gyrus. The results were visualized using the BrainNet Viewer package (http://www.nitrc.org/projects/bnv)

Using NBS, we identified a PKD‐related subnetwork with 19 nodes and 24 edges that was significantly altered in the PKD patients compared with HCs. The nodes included the following brain regions: bilateral caudate, bilateral pallidum, right thalamus, posterior cingulate gyrus, right hippocampus, bilateral medial superior frontal gyrus, right angular gyrus, right superior temporal gyrus, bilateral dorsolateral superior frontal gyrus, left middle frontal gyrus, right inferior frontal gyrus, bilateral superior parietal gyrus, left supramarginal gyrus, and right middle temporal gyrus (Figure 4). Significantly altered edges involving each of these regions were observed. All connectivity alterations within this network were decreased in the PKD group.

### 3.5 Relationships between network properties and clinical variables

**FIGURE 5:**
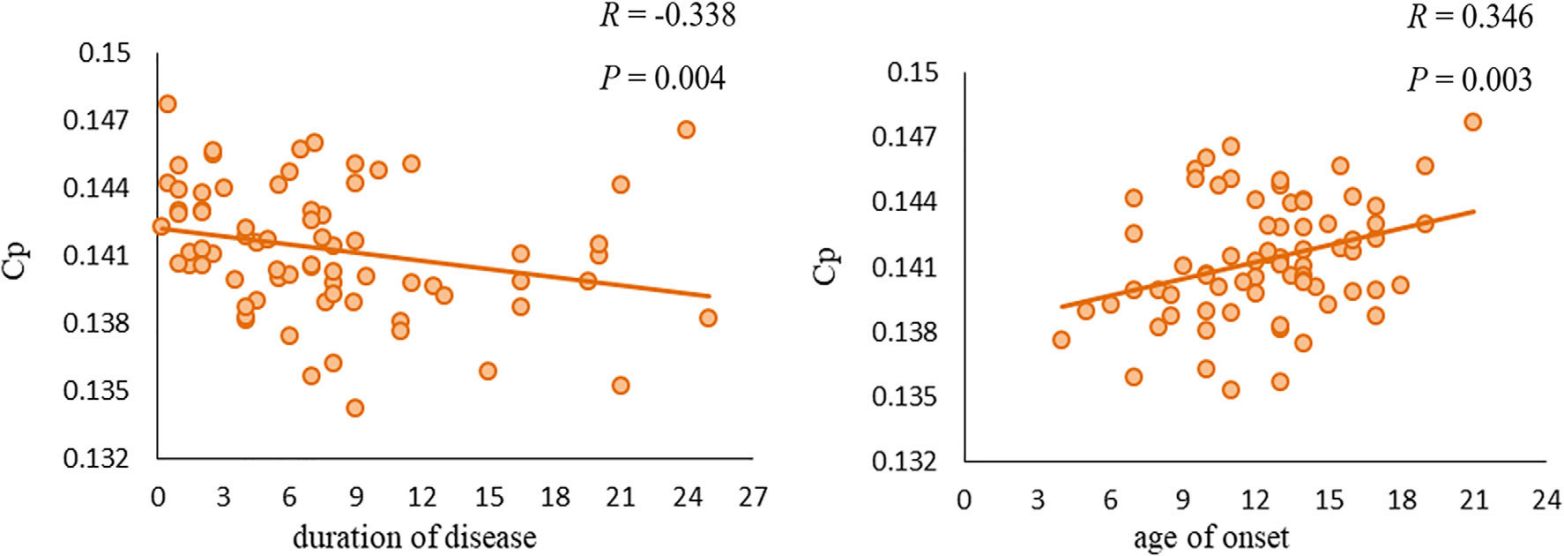
Scatter plots of the clustering coefficient (*Cp*) against the age of onset and duration of disease in patients with paroxysmal kinesigenic dyskinesia. *Cp* was negatively correlated with duration of disease (*r* = − .338, *p* = .004) and positively correlated with age of onset (*r* = .346, *p* = .003)

An outlier analysis was performed, and 14 patients were excluded because their age of onset, duration of disease, or because AUC values for network properties were less than Q1 − 3 (IQR) or more than Q3 + 3 (IQR) (where Q1 is the 25th percentile, Q3 is the 75th percentile, and the interquartile range (IQR) is Q3–Q1). After the outliers were excluded, the clustering coefficient (*Cp*) was negatively correlated with the duration of disease (*r* = −.338, *p* = .004) and positively correlated with the age of onset (*r* = .346, *p* = .003; Figure 5). There were no significant correlations between the clinical variables and any of the other global or nodal metrics. The results of the full correlation analysis, including the outliers, are given in Figure S2.

### 3.6 Single‐subject classification of patients and healthy controls

Using GM morphological network matrices, the mean balanced accuracy of classification was 87.8%, with sensitivity 87.6% and specificity 88.0% (*p* < .001), which is a mean balanced accuracy well above that expected by chance.

Having identified GM morphological network matrices as a powerful measure for detecting PKD, we examined the regions contributing to their superior performance. We computed the mean absolute value of the weights of the model across the different iterations of the cross‐validation. The 20 brain regions with the highest mean values are reported in Table 3; most of these were also among the regions with altered nodal properties.

**TABLE 3:** Top 20 most relevant brain regions for the classification analysis

No.	Regions
1	Lenticular nucleus, pallidum PAL.L
2	Inferior frontal gyrus, triangular part IFGtriang.R
3	Posterior cingulate gyrus PCG.R
4	Temporal pole: Middle temporal gyrus TPOmid.R
5	Caudate nucleus CAU.R
6	Angular gyrus ANG.L
7	Cuneus CUN.L
8	Temporal pole: Superior temporal gyrus TPOsup.R
9	Caudate nucleus CAU.L
10	Superior parietal gyrus SPG.L
11	Supramarginal gyrus SMG.L
12	Fusiform gyrus FFG.R
13	Middle frontal gyrus MFG.R
14	Inferior parietal, but supramarginal and angular gyri IPL.R
15	Thalamus THA.R
16	Inferior parietal, but supramarginal and angular gyri IPL.R
17	Inferior frontal gyrus, orbital part ORBinf.L
18	Superior frontal gyrus, medial SFGmed.R
19	Superior frontal gyrus, medial SFGmed.L
20	Lenticular nucleus, putamen PUT.L

*Note:* All brain regions are defined by AAL (automated anatomical labeling). Abbreviations as in Table 2.

## 4 DISCUSSION

The present study used graph theoretical analyses of structural MRI data to examine the topological organization of GM morphological networks in patients with PKD. There were three main findings: (i) at the global level (a) PKD patients showed decreased integration (reflected by increased *Lp*) and segregation (reflected by decreased *Cp*, *γ*, and *E*_loc_) in the organization of GM networks, a shift to a “weaker small‐worldness” pattern, and (b) the decrease in *Cp* was significantly correlated negatively with duration of disease and positively with age of onset; (ii) at the nodal level there were altered nodal characteristics in basal ganglia and thalamus, which are known to be involved in PKD; finally (iii) GM morphological network matrices could differentiate individual PKD patients from healthy individuals with high accuracy. These findings may provide new insights into the pathophysiology of PKD and aid development of new biomarkers for clinical diagnosis.

### 4.1 Global topological alterations in PKD patients

Both PKD patients and HCs showed high *Cp* and short *Lp*, demonstrating a shared small‐world topology in their GM morphological networks. However, relative to HCs, PKD patients showed higher *Lp* and lower *E*_loc_, *Cp*, *γ*, and *σ*, implying a disturbance in the normal small‐world organization. The brain's small‐world organization relates to an optimal balance between network integration (measured by *Lp*, *λ*, and *E*_glob_) and segregation (measured by *Cp*, *γ*, and *E*_loc_) of information processing (Bullmore & Sporns, 2012; Rubinov & Sporns, 2010; Suo et al., 2018). PKD patients had decreased global integration (increased *Lp*) and local segregation (decreased *Cp*, *γ*, and *E*_loc_) of their GM morphological networks, that is, a shift toward a “weaker small‐worldness” pattern according to the classification of Suo and colleagues (Suo et al., 2018). These results may relate to alterations of GM structure, for which there is some evidence in PKD: direct evidence from high‐resolution T1‐weighted MRI studies have identified morphometric/volumetric GM changes in presupplementary motor area, inferior frontal gyrus (H. F. Li et al., 2019) and thalamus (Kim et al., 2015); indirect evidence from secondary PKD reported that PKD was associated with various brain abnormalities for example, in thalamus (Camac, Greene, & Khandji, 1990), putamen (Merchut & Brumlik, 1986), right frontotemporal region (Gilroy, 1982), and globus pallidus (Micheli, Fernandez Pardal, Casas Parera, & Giannaula, 1986). Our morphological network findings are consistent with a structural brain network study using DTI, which showed “weaker small‐worldness” in PKD (L. Li et al., 2020). However, there was no significant change in functional global network properties in a study of drug‐naïve PKD patients using resting‐state functional MRI (Y. Zhang et al., 2020). These differences might be due to the different imaging modalities. Functional MRI–derived networks characterize synchronized brain activity at a point in time, while structural networks reflect more stable patterns of anatomical organization affected by heredity, experience‐related plasticity, and mutually trophic reinforcement (Alexander‐Bloch et al., 2013; Kong et al., 2015). Future studies should employ a multimodal approach.

### 4.2 Nodal topological alterations in PKD patients

In addition to changes in global network characteristics, specific nodal changes were observed in the basal ganglia (caudate nucleus and pallidum) and thalamus, which have been shown to be involved in PKD (Joo et al., 2005; Thiriaux et al., 2002; Zhou, Chen, Gong et al., 2010). There are two pathways in the basal ganglia circuitry. The striatum (putamen and caudate) sends an inhibitory projection to the basal ganglia output nuclei (globus pallidus interna/substantia nigra, Gpi/SNr), called the direct pathway. The striatum also sends an inhibitory projection to the globus pallidus externa (Gpe), which, in turn, inhibits the subthalamic nucleus (STN) and Gpi. This latter is known as the indirect pathway and acts in opposition to the direct pathway. The Gpi/SNr sends inhibitory fibers to the thalamus, and the thalamus, in turn, projects excitatory fibers to the motor cortex and supplementary motor cortex, which project to the spinal motor neurons, controlling muscle contractions and movements (Breakefield et al., 2008; Peterson, Sejnowski, & Poizner, 2010). It is also widely accepted that PKD is a paroxysmal movement disorder caused by abnormalities of the basal ganglia‐thalamo‐cortical circuit. There is evidence that PKD may be associated with structural and functional abnormalities in the basal ganglia and thalamus regions (Joo et al., 2005; Kim et al., 2015; Shirane et al., 2001; Zhou, Chen, Gong, et al., 2010; Zhou, Chen, Zhang, et al., 2010). However, at present there is no consensus on which subcortical GM structure within the basal ganglia‐thalamo‐cortical network is primarily responsible for the abnormal involuntary movements. Based on our findings of nodal changes in the caudate nucleus, pallidum, and thalamus, we suggest that abnormalities in both the basal ganglia and the thalamus are primary contributors to abnormal motor symptoms in patients with PKD.

We also found decreased nodal centralities in bilateral caudate nucleus (implying weaker information transfer and integration) and increased nodal centralities in bilateral pallidum and thalamus (stronger information transfer and integration). These results are broadly consistent with the hypothesis that loss of normal inhibitory control of basal ganglia and thalamus output, and the resulting overexcitation of the thalamocortical circuit, underlie the hyperkinetic movement of PKD (Mink, 2003; Quartarone & Hallett, 2013; Y. Zhang et al., 2020).

Altered nodal metrics in PKD were also found in posterior cingulate gyrus, right hippocampus, bilateral medial superior frontal gyrus, right angular gyrus, and right superior temporal gyrus. These regions are the vital regions of the default‐mode network (DMN) (Buckner, Andrews‐Hanna, & Schacter, 2008; Montembeault, Rouleau, Provost, & Brambati, 2016). Although DMN abnormalities are not commonly reported in PKD, they have been described in other hyperkinetic movement disorders, such as writer's cramp (Mohammadi et al., 2012) and Tourette syndrome (Wen et al., 2017). Further, altered nodal centralities in posterior cingulate gyrus, angular gyrus, and temporal pole have been reported in the functional network of drug‐naïve PKD patients (Y. Zhang et al., 2020). One critical function of the DMN is in emotional processing (Mohan et al., 2016). PKD symptoms depend on the internal emotional state: anxiety and stress are known to lower the threshold for attacks in many PKD patients (Bruno et al., 2004; De Gusmao & Silveira‐Moriyama, 2019). The attack can also be triggered by startle (Waln & Jankovic, 2015). Moreover, patients with PKD exhibit manifestations of anxiety or depression disorders (Kunii, Matsuda, & Yabe, 2017; Tian et al., 2017). Our findings may help account for this, defining structural network abnormalities that may underlie abnormal emotional processing in PKD.

Interestingly, we observed nodal changes in the bilateral dorsolateral superior frontal gyrus, left middle frontal gyrus, right inferior frontal gyrus, bilateral superior parietal gyrus, and left supramarginal gyrus which are the key regions of the central executive network (CEN) (Menon, 2011; Niu et al., 2018; Patel, Spreng, Shin, & Girard, 2012; Sylvester et al., 2012). The CEN plays a crucial role in the execution, control, and inhibition of task performance (Y. Chen et al., 2016; Littow et al., 2015). Most PKD patients have a sensory aura which alerts them to try to control or suppress the attacks by ceasing general movement and performing determined movements with the affected limb, but only a few succeed in aborting or minimize the attack (Bruno et al., 2004; Silveira‐Moriyama et al., 2013). Our finding of decreased node centralities in most CEN regions may reflect this impairment. Furthermore, attacks in PKD are frequently unilateral and asymmetric, and patients often try to control the affected limb with the normal limb (De Gusmao & Silveira‐Moriyama, 2019). Our finding of increased nodal centralities in left dorsolateral superior frontal gyrus, the cardinal region in CEN, may reflect the brain's adaptive plasticity, given that PKD patients must expend more effort to inhibit attacks. However, our novel demonstration of nodal changes in CEN requires further study.

### 4.3 Significant relations between global network properties and clinical variables

We found that alterations in global network properties in PKD are associated with illness duration and age of onset. In particular, the decreased *Cp* was significantly correlated negatively with disease duration and positively with the age of onset. Some previous studies in PKD failed to detect significant correlations between outcomes and illness duration (Kim et al., 2015; Zhou, Chen, Gong, et al., 2010; Zhou, Chen, Zhang, et al., 2010). However, Long et al. (2017) found that the functional connectivity (FC) of the thalamo–motor‐cortical network was positively correlated with disease duration, and a recent study also reported a negative correlation of disease duration with GM volume in the presupplementary motor area (H. F. Li et al., 2019). Our results are consistent with this, suggesting that with greater illness duration the segregation (reflected by *Cp*) of GM morphological networks decreased. Although few studies have reported the relation between outcomes and age of onset in PKD, a recent functional network study found significant correlations between nodal efficiency of left pallidum and the age of onset in patients with PKD (Y. Zhang et al., 2020). And we found positive correlations between decreased *Cp* and age of onset, suggesting that the earlier the onset, the more the decrease in the segregation function of the GM morphological networks. Of course we cannot say whether the changes in *Cp* are the cause or consequence of repeated movement symptoms in PKD. More studies are needed to clarify this.

### 4.4 The classifying ability of GM morphological network matrices

Modern neuroimaging in conjunction with machine learning is particularly suited to the study of complex neuropsychiatric diseases. In the SVM analysis, we achieved a high (87.8%) classification accuracy using GM morphological network matrices, showing that GM morphological network matrices were sensitive in identifying PKD from controls. Our results provide support for the emerging view that GM structural networks, which capture cellular, molecular and functional features of the brain, are a powerful tool to examine the structural organization of psychiatric and neurological illnesses (T. Chen et al., 2017; Niu et al., 2018; Seidlitz et al., 2018; Tijms et al., 2014; Yun et al., 2020), and that network biomarkers which can capture the role of brain network structure in a given phenotype, and study the role of known subsystems in a particular disorder, have the potential to improve diagnosis of neuropsychiatric diseases (Schindlbeck & Eidelberg, 2018; Wen et al., 2017). In particular, a recent study suggested that connectome‐wide matrices had greater diagnostic value than graph‐based metrics or preprocessed whole‐brain image data (Lei et al., 2019). Our results are consistent with this, suggesting that GM morphological network matrices (i.e., connectome‐wide matrices) might have high diagnostic value for PKD. Moreover, we found that the 20 brain regions that provided the greatest contribution to the classification overlapped with the regions known to have altered nodal properties in PKD. Importantly, these included the caudate nucleus, pallidum, and thalamus, which are well known to be altered in PKD. This further supports the concept that GM morphological network matrices might serve as a neuroimaging biomarker to assist the clinical diagnosis of PKD.

### 4.5 Limitations

The study has several limitations. First, different templates may cause considerable variations in graph‐based theoretical parameters (J. Wang et al., 2009). We tested the reproducibility of our findings by constructing brain networks based on an alternative templates (the Harvard‐Oxford atlas), and found similar results (Table S1). However, more templates are needed to test the reproducibility of the results in the future. Second, most patients were treated with AEDs. Although we recruited only patients who had been on medication for <3 months or had not taken medication within the 6 months prior to the study, most were taking a low dose of AEDs to control the attacks and avoid the side effects (Huang et al., 2015). The treated patients were scanned only after medication had been withdrawn for at least 12 hr, and there were no significant correlations between the topological metrics (AUC) and months on medication within the PKD group. Nonetheless, we cannot completely eliminate the effects of AEDs. Third, the recruited PKD patients were heterogeneous in terms of genetic mutation. Although the precise role of proline‐rich transmembrane protein 2 (PRRT2) mutations in the pathophysiology of PKD remains unknown, a subgroup analysis should be conducted to address whether the PRRT2‐mutattion specifically affects GM morphological networks. Fourth, the biological significance of these network alterations needs to be more fully understood. Although accumulating evidence indicates that heredity, experience‐related plasticity, mutually trophic influences and coordinated neurodevelopmental and aging trajectories play important roles in the formation of morphological brain networks (Alexander‐Bloch et al., 2013; Evans, 2013), we need to clarify which of these factors is related to the abnormalities in PKD. Fifth, although the methodology we used (Kong et al., 2014; Kong et al., 2015) to extract individual structural morphology brain networks is completely data‐driven, the relatively wide age range of participants might have biased the results. We therefore performed a subgroup analysis by dividing the sample (patients and controls) into adult and adolescents groups, and analyzing separately: the results were similar to our main findings (Tables S2‐S5).

## 5 CONCLUSION

Our study provides the first demonstration that PKD features disrupted topological organization in GM morphological networks. Patients with PKD also showed altered nodal properties in basal ganglia and thalamus, which were previously shown to be involved in the disease. Furthermore, the GM morphological network matrices permitted differentiation of PKD from healthy controls with a high accuracy of 87.8%, indicating that GM morphological network matrices were sensitive in identifying PKD from controls. Our results provide structural insights into the brain networks associated with PKD and may help extend our understanding of how structural disruptions of GM networks are linked to the pathophysiology of PKD. GM morphological network matrices also have potential as neuroimaging biomarkers to assist in clinical PKD diagnosis.

## CONFLICT OF INTEREST

All authors declare no competing interests.

## Supporting information


**Appendix** **S1:** Supporting Information

## Data Availability

The data that support the findings of this study are available from the corresponding author upon reasonable request. The data and code sharing protocols adopted by the authors comply with the requirements of the funding institute, and comply with institutional ethics approval.
