# Supplementary material for: Disruption of gray matter morphological networks in patients with paroxysmal kinesigenic dyskinesia
Source: Hum Brain Mapp. 2020 Oct 15;42(2):398–411. doi: 10.1002/hbm.25230 (PMC7776009; doi:10.1002/hbm.25230)

**Supporting Information**

**1. Lack of dependence on brain parcellation template**

Different brain parcellation schemes may yield different topological organizations for the resultant brain networks (Sanabria-Diaz et al., 2010; Wang et al., 2009). To determine whether our main results were dependent on the choice of parcellation template, we repeated the analysis using the Harvard-Oxford atlas, which divides the brain into 112 regions (Kennedy et al., 1998; Makris et al., 1999).

Using the Harvard-Oxford atlas, we found that both the PKD and control groups exhibited small-world topology (σ > 1). Compared with HCs, the PKD patients showed a significantly increased characteristic path length (Lp; p = 0.032) and a decreased global efficiency (Eglob; p = 0.002), clustering coeﬃcient (Cp; p = 0.049), and normalized clustering coefficient (γ; p = 0.023). No significant difference was identified in any other global efficiency. We identified brain regions that exhibited significant between-group differences in at least one nodal metric (false discovery rate corrected at a significance level of .05). Compared with HCs, the PKD patients exhibited increased node centrality in the left superior frontal gyrus, right middle frontal gyrus, right posterior cingulate gyrus, left paracingulate gyrus, right hippocampus, right precuneus, and bilateral pallidum. The PKD patients exhibited decreased nodal centrality in the right superior frontal gyrus, left middle frontal gyrus, bilateral inferior frontal gyrus (triangular part), left superior parietal lobule, left supramarginal gyrus (anterior division), right postcentral gyrus, right angular gyrus, left caudate, right superior temporal gyrus (anterior division), right middle temporal gyrus (anterior division), and left parietal opercular cortex (Table S1). These findings are similar to our main results.

References:

Kennedy, D. N., Lange, N., Makris, N., Bates, J., Meyer, J., & Caviness, V. S., Jr. (1998). Gyri of the human neocortex: an MRI-based analysis of volume and variance. Cereb Cortex, 8(4), 372-384. https://doi.org/10.1093/cercor/8.4.372

Makris, N., Meyer, J. W., Bates, J. F., Yeterian, E. H., Kennedy, D. N., & Caviness, V. S. (1999). MRI-Based topographic parcellation of human cerebral white matter and nuclei II. Rationale and applications with systematics of cerebral connectivity. Neuroimage, 9(1), 18-45. https://doi.org/10.1006/nimg.1998.0384

Sanabria-Diaz, G., Melie-Garcia, L., Iturria-Medina, Y., Aleman-Gomez, Y., Hernandez-Gonzalez, G., Valdes-Urrutia, L., . . . Valdes-Sosa, P. (2010). Surface area and cortical thickness descriptors reveal different attributes of the structural human brain networks. Neuroimage, 50(4), 1497-1510. https://doi.org/10.1016/j.neuroimage.2010.01.028

Wang, J., Wang, L., Zang, Y., Yang, H., Tang, H., Gong, Q., . . . He, Y. (2009). Parcellation-dependent small-world brain functional networks: a resting-state fMRI study. Hum Brain Mapp, 30(5), 1511-1523. https://doi.org/10.1002/hbm.20623

**Table S1.** Regions showing altered node centrality in PKD patients compared to HC participants analyzed using the Harvard-Oxford atlas

| Brain regions | p values | | |
| --- | --- | --- | --- |
|  | Nodal betweenness | Nodal degree | Nodal efficiency |
| **PKD>HC** |  |  |  |
| L superior frontal gyrus | 0.0016* | 0.0248 | 0.0153 |
| R middle frontal gyrus | 0.0353 | 0.0004* | 0.0011* |
| R posterior cingulate gyrus | 0.1278 | 0.0007* | 0.0344 |
| L paracingulate gyrus | 0.0182 | 0.0014* | 0.0031* |
| R hippocampus | < 0.0001* | < 0.0001* | 0.0058 |
| R precuneus | 0.0079 | 0.0001* | 0.0461 |
| L pallidum | 0.0627 | 0.0004* | 0.0011* |
| R pallidum | < 0.0001* | 0.0001* | 0.0533 |
| **PKD<HC** |  |  |  |
| R superior frontal gyrus | < 0.0001* | 0.4350 | 0.0019* |
| L middle frontal gyrus | 0.3230 | 0.0002* | 0.0008* |
| L inferior frontal gyrus (triangular part) | 0.3920 | 0.0078 | < 0.0001* |
| R inferior frontal gyrus (triangular part) | < 0.0001* | 0.0007* | 0.0022* |
| L superior parietal lobule | 0.0831 | < 0.0001* | 0.0008* |
| L supramarginal gyrus (anterior division) | 0.0114 | < 0.0001* | 0.0004* |
| R postcentral gyrus | 0.7125 | 0.0016* | < 0.0001* |
| R angular gyrus | 0.0016* | 0.0149 | 0.0027* |
| L caudate | < 0.0001* | 0.0563 | < 0.0001* |
| R superior temporal gyrus (anterior division) | 0.0111 | 0.0087 | < 0.0001* |
| R middle temporal gyrus (anterior division) | < 0.0001* | < 0.0001* | < 0.0001* |
| L parietal opercular cortex | 0.0074 | < 0.0001* | 0.0093 |

The regions listed above are those that exhibited significant between-group differences in at least one node centrality parameter (marked by an asterisk). Benjamini-Hochberg false discovery rate correction was applied to each nodal measure. All p values were obtained by using a permutation test. All the brain regions were defined by Harvard-Oxford atlas. Abbreviations: PKD: paroxysmal kinesigenic dyskinesia; HC: healthy controls; R: right; L: left

**2. Possible age effects**

Because of the rather wide age-range of our patient group, we also analyzed separately adult (≥18 years) and adolescent (< 18 years) PKD patients, comparing each with an age-matched subset of HCs. The results are presented below in Tables S2-S5.

**Table S2.** Group differences in global brain network properties between adult PKD patients (≥18 years) and healthy controls.

| Global  measurements | PKD (n=58)  Mean ± SD | HC (n=84)  Mean ± SD | *p* values |
| --- | --- | --- | --- |
|
| E_glob_ | 0.1064 ± 0.0018 | 0.1064 ± 0.0015 | 0.4848 |
| E_loc_ | 0.1691 ± 0.0029 | 0.1702 ± 0.0023 | 0.0084* |
| Cp | 0.1409 ± 0.0028 | 0.1416 ± 0.0025 | 0.0663 |
| L_p_ | 0.5594 ± 0.0111 | 0.5536 ± 0.0095 | 0.0484* |
| γ | 0.4203 ± 0.0289 | 0.4341 ± 0.0293 | 0.0020* |
| λ | 0.2766 ± 0.0044 | 0.2765 ± 0.0036 | 0.4110 |
| **σ** | 0.3623 ± 0.0250 | 0.3743 ± 0.0254 | 0.0031* |

Significant difference of topological metrics between groups at p < 0.05 was marked by asterisk. Abbreviations: Eglob: global efficiency; Eloc: local efficiency; Cp: clustering coeﬃcient; Lp: characteristic path length; γ: normalized clustering coefficient; λ: normalized characteristic path length; σ: small-worldness; HC: healthy controls; PKD: paroxysmal kinesigenic dyskinesia; SD: standard deviation.

**Table S3.** Regions showing altered node centrality in adults PKD patients (≥18 years) and healthy controls

| Brain regions | *p values* | | |
| --- | --- | --- | --- |
|  | Nodal betweenness | Nodal degree | Nodal efficiency |
| **PKD>HC** |  |  |  |
| L superior frontal gyrus, dorsolateral | 0.0002* | 0.4763 | 0.1045 |
| R posterior cingulate gyrus | < 0.0001* | 0.0737 | 0.0007* |
| R hippocampus | < 0.0001* | 0.0788 | 0.4771 |
| R superior occipital gyrus | 0.0032* | 0.4048 | 0.4576 |
| L pallidum | 0.0533 | 0.0004* | < 0.0001* |
| R pallidum | 0.2047 | 0.0496 | < 0.0001* |
| L thalamus | < 0.0001* | 0.1279 | 0.1222 |
| **PKD<HC** |  |  |  |
| R inferior frontal gyrus, triangular part | 0.4387 | < 0.0001* | 0.0002* |
| L superior frontal gyrus, medial | 0.2409 | 0.0060 | 0.0004* |
| R superior frontal gyrus, medial | 0.0109 | 0.0001* | 0.0004* |
| L superior parietal gyrus | 0.3477 | 0.0026* | 0.0060* |
| R superior parietal gyrus | 0.2113 | 0.0062 | 0.0005* |
| L angular gyrus | 0.0495 | 0.0055 | 0.0046* |
| L caudate | 0.0319 | < 0.0001* | < 0.0001* |
| R caudate | 0.4278 | < 0.0001* | < 0.0001* |
| R temporal pole, superior temporal gyrus | 0.0284 | < 0.0001* | < 0.0001* |
| R temporal pole, middle temporal gyrus | 0.1455 | 0.0002* | 0.0003* |

These regions exhibited significant between-group differences in at least one node centrality parameter (marked by asterisk). Benjamini-Hochberg false discovery rate correction was applied to each nodal measure. All p values were obtained using a permutation test (10,000 permutations). All the brain regions were defined by AAL (automated anatomical labeling). Abbreviations: PKD: paroxysmal kinesigenic dyskinesia; HC: healthy controls; R: right; L: left

**Table S4.** Group differences in global brain network properties between adolescent PKD patients (< 18 years) and healthy controls.

| Global  measurements | PKD (n=29)  Mean ± SD | HC (n=31)  Mean ± SD | *p* values |
| --- | --- | --- | --- |
|
| E_glob_ | 0.1057 ± 0.0021 | 0.1061 ± 0.0018 | 0.2125 |
| E_loc_ | 0.1692 ± 0.0034 | 0.1708 ± 0.0028 | 0.0249* |
| Cp | 0.1412 ± 0.0032 | 0.1428 ± 0.0025 | 0.0373* |
| L_p_ | 0.5630 ± 0.0125 | 0.5543 ± 0.0107 | 0.0362* |
| γ | 0.4242 ± 0.0309 | 0.4262 ± 0.0302 | 0.3933 |
| λ | 0.2774 ± 0.0044 | 0.2781 ± 0.0045 | 0.2925 |
| σ | 0.3644 ± 0.0257 | 0.3658 ± 0.0275 | 0.4092 |

Significant difference of topological metrics between groups at p < 0.05 was marked by asterisk. Abbreviations: Eglob: global efficiency; Eloc: local efficiency; Cp: clustering coeﬃcient; Lp: characteristic path length; γ: normalized clustering coefficient; λ: normalized characteristic path length; σ: small-worldness; HC: healthy controls; PKD: paroxysmal kinesigenic dyskinesia; SD: standard deviation.

**Table S5.** Regions showing altered node centrality between adolescent PKD patients (< 18 years) and healthy controls.

| Brain regions | *p values* | | |
| --- | --- | --- | --- |
|  | Nodal betweenness | Nodal degree | Nodal efficiency |
| **PKD>HC** |  |  |  |
| R middle frontal gyrus, orbital part | 0.4381 | 0.0014* | 0.0031* |
| R inferior frontal gyrus, triangular part | 0.3650 | 0.0046* | 0.0012* |
| L parahippocampal gyrus | 0.0982 | 0.0006* | 0.0005* |
| L supplementary motor area | 0.0235 | 0.0915 | 0.0025* |
| R calcarine fissure and surrounding cortex | 0.0052 | 0.0039* | 0.0075 |
| L pallidum | 0.0029* | 0.0015* | 0.0004* |
| R pallidum | 0.2336 | 0.3328 | 0.0003* |
| L thalamus | < 0.0001* | 0.1283 | 0.1313 |
| **PKD<HC** |  |  |  |
| L superior frontal gyrus, medial | 0.2910 | 0.0045* | 0.0062 |
| L superior parietal gyrus | 0.2365 | 0.0038* | 0.0040* |
| L inferior parietal, but supramarginal and angular gyri | 0.0222 | 0.0049* | 0.0024* |
| L caudate | 0.0442 | 0.0032* | 0.0026* |
| R caudate | 0.4875 | <0.0006* | < 0.0036* |
| R Temporal pole: superior temporal gyrus | 0.0415 | 0.0037* | 0.0158 |
| R temporal pole, middle temporal gyrus; | 0.4454* | 0.0010* | 0.0006* |

These regions exhibited significant between-group differences in at least one node centrality parameter (marked by asterisk). Benjamini-Hochberg false discovery rate correction was applied to each nodal measure. All p values were obtained using a permutation test (10,000 permutations). All the brain regions were defined by AAL (automated anatomical labeling). Abbreviations: PKD: paroxysmal kinesigenic dyskinesia; HC = healthy controls; R = right; L = left

**3. Both PKD and HC exhibit small-world topology in the gray matter morphological network**

**Figure S1.** Graph shows the key parameters, normalized clustering coefficients (Cp) and normalized path lengths (Lp), as a function of chosen sparsity

**
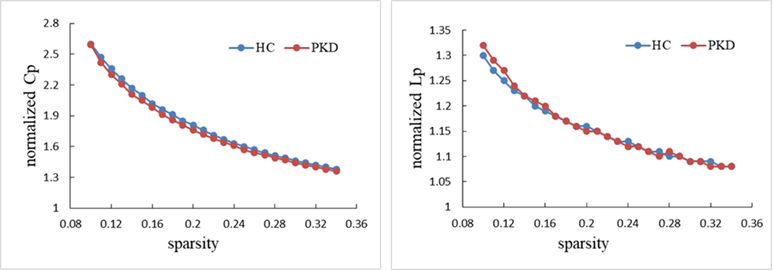
**

In the defined threshold range, both the PKD and control groups exhibited normalized clustering coefficients (Cp) substantially larger than 1 and normalized path lengths (Lp) approximately equal to 1, the typical features of small-world topology. Abbreviations: HC = healthy control; PKD = paroxysmal kinesigenic dyskinesia.

**4. The results of correlation analysis without excluding outliers**

We found that the duration of disease was negatively correlated with Cp (r = -0.324, p = 0.003), Eloc (r = -0.241, p = 0.027), and γ (r = -0.236, p = 0.031) but not with the other global metrics and the age of onset was positively correlated with Cp (r = 0.326, p = .002), Eloc (r = 0.244, p = 0.025), and γ (r = 0.238, p = 0.029) but not with the other global metrics. We did not observe any significant correlations between the clinical variables and the nodal metrics. The results are shown in Figure S2.

**Figure S2**. Scatter plots of age of onset and duration of disease against the global metric (Cp, Eloc, and γ)


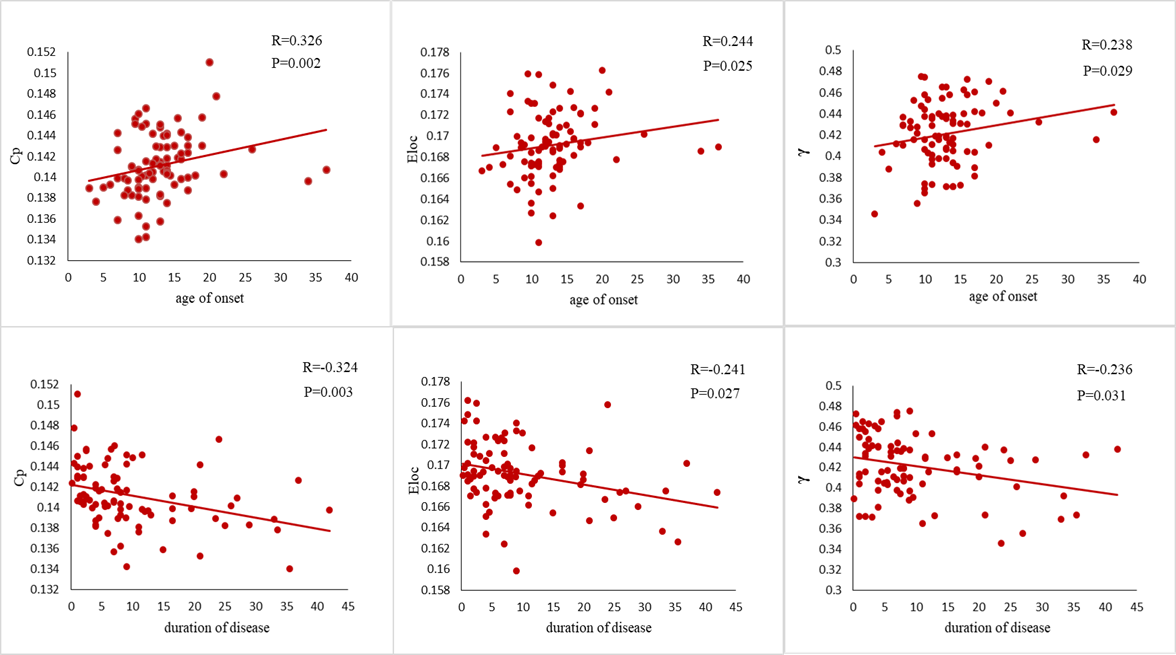

Supplement: Supplementary file 1 — Appendix S1: Supporting Information [file HBM-42-398-s001.doc]
